# O.4.1-7 Recreational football training increases leg-extensor force production at high velocities in 55- to 70-year-old adults

**DOI:** 10.1093/eurpub/ckad133.172

**Published:** 2023-09-11

**Authors:** Evelien Van Roie, Chiel Poffé, Arne Jaspers, Filip Boen, Werner Helsen, Katrien Koppo

**Affiliations:** Physical Activity, Sports & Health Research Group, Department of Movement Sciences, KU Leuven, Belgium; Exercise Physiology Research Group, Department of Movement Sciences, KU Leuven, Belgium; Research Group for Musculoskeletal Rehabilitation, Department of Rehabilitation Sciences, KU Leuven, Belgium; Physical Activity, Sports & Health Research Group, Department of Movement Sciences, KU Leuven, Belgium; Movement Control and Neuroplasticity Research Group, Department of Movement Sciences, KU Leuven, Belgium; evelien.vanroie@kuleuven.be; Exercise Physiology Research Group, Department of Movement Sciences, KU Leuven, Belgium

## Abstract

**Purpose:**

Ageing coincides with a blunted ability to produce force at moderate to high velocities. Therefore, improving muscle power, especially at moderate to high velocities, is a crucial target for exercise interventions in middle-aged and older adults. We investigated the effects of 10 weeks of recreational football training on the leg-extensor force-velocity (F-V) profile in 55- to 70-year-old adults. Simultaneous effects on functional capacity, body composition and endurance exercise capacity were examined. In addition, feasibility and the physical demands of the training program were tracked.

**Methods:**

Forty participants (age 63.5 ± 3.9 years) were randomized in a football training (FOOT, n = 20) and a control (CON, n = 20) group. FOOT performed 45-min to 1-h of football training sessions with small-sided games (SSG’s) twice a week. Pre and post intervention, leg-extensor F-V profile (maximal power (P_max_), maximal force (F_0_), maximal velocity (V_0_), the slope divided by F_0_ (S_FV_/F_0_)), functional capacity, body composition and endurance exercise capacity were measured.

**Results:**

Results revealed an increase over time for P_max_ (p_time_ = 0.009), but no interaction effect (p_int_ = 0.221). A greater increase in V_0_ and in S_FV_/F_0_ was apparent in FOOT compared to CON (p_int_ = 0.043 and p_int_ = 0.065). No interaction effect was found for F_0_ (p_int_ = 0.922). Three-step stair ascent power, 10m fast walk, body fat percentage and running speed at 2mM lactate improved significantly more in FOOT than in CON (all p_int_ < 0.1). The SSG’s elicited intense muscular actions and high average heart rates of 85.7% of maximal heart rate. Despite the high training load, participants perceived the sessions as very enjoyable and feasible.

**Conclusions:**

The present study emphasized that recreational football can be used as a feasible, enjoyable and effective training tool in middle-aged to older adults for improving force production at high velocities. This improvement is translated into a better performance on functional capacity tests that rely on high execution velocity. In addition, broad-spectrum health benefits can be realized with only 2 hours of training per week.

**Funding:**

E. Van Roie was supported by the Research Foundation Flanders, Belgium (senior postdoctoral fellowship 12Z5720N).

